# Crystal structure and fluorescence study of (μ-*N*-[(3,5-dimethyl-1*H*-pyrrol-2-yl)methyl­idene]-*N*-{4-[(3,5-dimethyl-1*H*-pyrrol-2-yl)methyl­idene­aza­nium­yl]phen­yl}aza­nium)bis­[di­fluorido­boron(IV)]

**DOI:** 10.1107/S2056989021000463

**Published:** 2021-01-15

**Authors:** Xiaoxue Liu, Tuo Li, Zhenming Yin

**Affiliations:** a College of Chemistry, Tianjin Key Laboratory of Structure and Performance for Functional Molecule, Tianjin Normal University, Tianjin 300387, People’s Republic of China

**Keywords:** 2-imino­pyrrole, BF_2_ complex, crystal structure, fluorescence

## Abstract

The title mol­ecule, C_20_H_20_B_2_F_4_N_4_, assumes a planar conformation with all atoms apart from the F atoms lying on the symmetry plane. Each boron atom is four-coordinated by two fluorine atoms, a pyrrole N atom and an imine N atom. Both imine CH=N groups adopt a *trans* conformation. In the crystal, the mol­ecules self-assemble into a pillar structure through C—H⋯F hydrogen bonds and π–π inter­actions. The UV–vis spectrum and fluorescence spectra of the title compound are also reported.

## Abstract 2

In the title compound, each boron atom is four-coordinated by two fluorine atoms, a pyrrole N atom and an imine N atom. Both imine CH=N groups adopt a *trans* conformation. In the crystal, the mol­ecules self-assemble into a pillar structure through C—H⋯F hydrogen bonds and π–π inter­actions.

## Chemical context   

Fluorescent materials are gradually becoming a necessity in modern chemistry and biology because of their unique advantages in the characterization of life activities in living organisms (Zhang *et al.*, 2019 ▸). Boron-dipyrromethene (BODIPY) is a frequently reported fluorescent structure (Boens *et al.*, 2015 ▸). Its planar structure endows BODIPY compounds with strong fluorescence emission under the action of excitation light. Such compounds also have high molar absorption coefficient, good light stability and excitation wavelengths in the visible to near infrared region. In addition, their structures can easily be modified and they are not easily affected by the environment (Loudet & Burgess, 2007 ▸). The success of BODIPY dyes has led to research on similar structures such as aza-BODIPY structures (Bodio & Goze, 2019 ▸), boron complexes of imino­pyrrolide ligands (BOIMPY; Suresh *et al.*, 2012 ▸, 2015 ▸; Lee *et al.*, 2016 ▸), bis­(di­fluoro­boron)-1,2-bis­{(pyrrol-2-yl)methyl­ene}hydrazine (BOPHY; Boodts *et al.*, 2018 ▸) structures and other novel organoboron fluorescence materials (Frath *et al.*, 2014 ▸).

BOIMPY has a similar structure to BODIPY, in which the pyrrole ring is located in the same plane as the aromatic ring, the boron atom and the methyl­ene bridge. More importantly, BOIMPY has the advantage of lower mol­ecular symmetry, which can overcome the shortcoming of the short Stokes shifts of BODIPY (Lee *et al.*, 2016 ▸). In contrast to BODIPY, studies on BOIMPY are still rare. Herein, we report the synthesis, crystal structure and spectroscopic properties of a new BOIMPY compound, bis­(di­fluoro­boron)bis­(pyrrol-2-yl)meth­yl­enedi­amino­phenyl­ene.

## Structural commentary   

**Figure 1:**
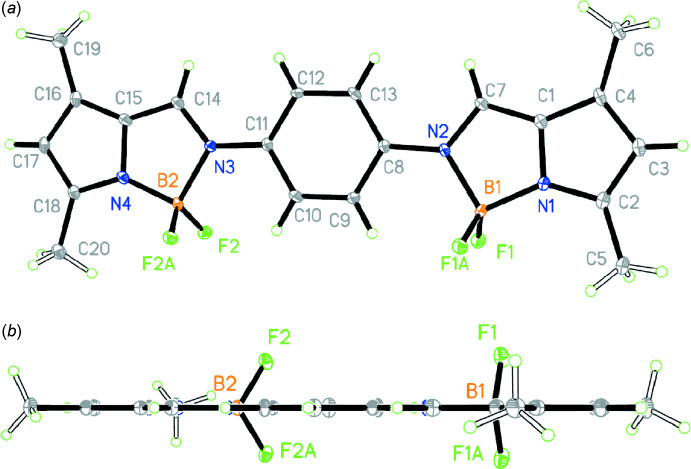
*ORTEP* diagrams for the title compound, (*a*) top view and (*b*) side view, with displacement ellipsoids drawn at the 30% probability level.

The structure of the title compound is shown in Fig. 1 ▸. All atoms lie on the symmetry plane except for the F atoms, which deviate from it by 1.136 (1) Å (F1) and 1.135 (1) Å (F2) on the same side of the mol­ecule. Each boron atom is four-coordinated by two fluorine atoms, a pyrrole N atom and an imine N atom. The N1—B1, N2—B1, N3—B2 and N4—B2 bond lengths [1.544 (4), 1.604 (4), 1.610 (4) and 1.538 (4) Å, respectively] are longer than the accepted mean value for a B—N bond (1.54–1.55Å) in BODIPY compounds reported in the literature (Madhu & Ravikanth, 2014 ▸). The two imine CH=N groups adopt a *trans* conformation and at 1.339 (4) and 1.321 (4) Å their bond lengths are longer than that in the free imino-pyrrole ligand (1.263 Å; Xu *et al.*, 2010 ▸) while the C8—N2 and C11—N3 bonds [both 1.408 (4) Å] are shorter than in the free imino-pyrrole ligand (1.424 Å; Xu *et al.*, 2010 ▸).
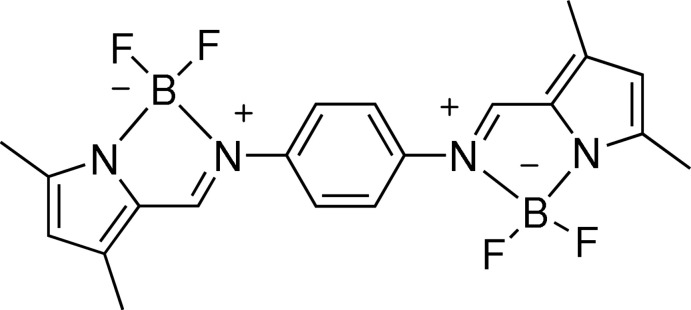


## Supra­molecular features   

**Figure 2:**
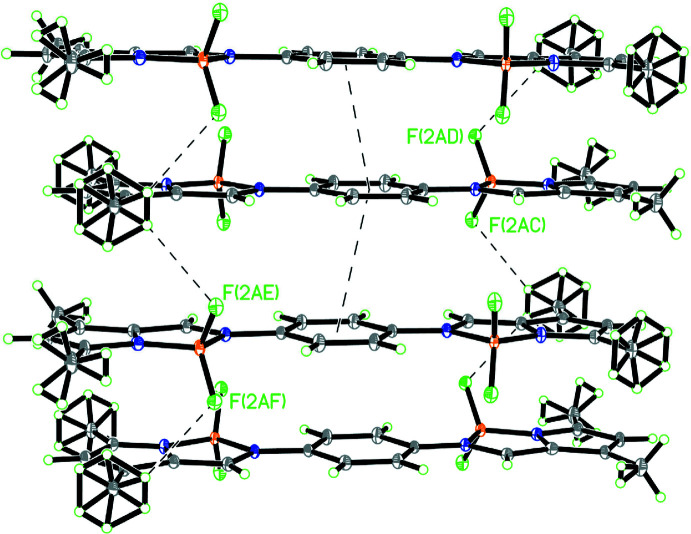
Part of the pillar structure showing mol­ecules linked by C—H⋯F^i^ hydrogen bonds and π–π inter­action [symmetry code: (i) −*x* + 1, *y* + 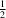, −*z* + 1].

**Figure 3:**
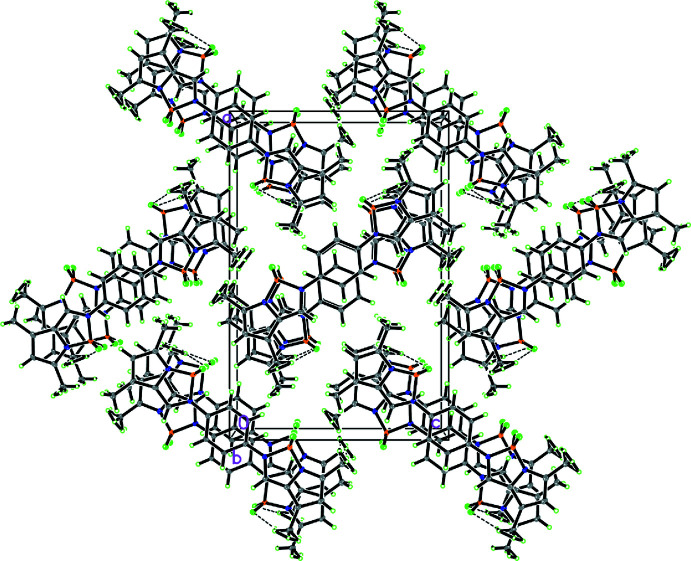
Part of the packing diagram for the title compound.

In the crystal, the mol­ecules are linked by C6—H6*B*⋯F2 hydrogen bonds between methyl group and the fluorine atom (Table 1 ▸), and π–π inter­actions between benzene rings [*Cg*1⋯*Cg*1(−*x* + 1, *y* + 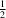, −*z* + 1) = 3.7521 (2) Å; *Cg*1 is the centroid of the C8–C13 ring] into one-dimensional pillars along the *b*-axis direction. Within the pillar, neighbouring mol­ecules are oriented in opposite directions (Fig. 2 ▸). The pillars are held together by van der Waals inter­actions, forming a herringbone structure. A perspective view of the crystal packing within the unit cell is depicted in Fig. 3 ▸.

## Database survey   

A search in the Cambridge Structural Database (CSD, version 5.41, update of November 2019; Groom *et al.*, 2016 ▸) returned 21 entries for imino­pyrrolyl boron complexes. Two di­phenyl­boron analogues of the title compound were reported by Gomes and coworkers [KEDHIM (Suresh *et al.*, 2012 ▸) and TUJFOV (Suresh *et al.*, 2015 ▸)]. In their crystals, the respective dihedral angles between the 2-formimino­pyrrolyl unit and the phenyl ring are −47.2 (3) and 46.1 (11)°.

## UV–vis spectrum and fluorescence spectra   

**Figure 4:**
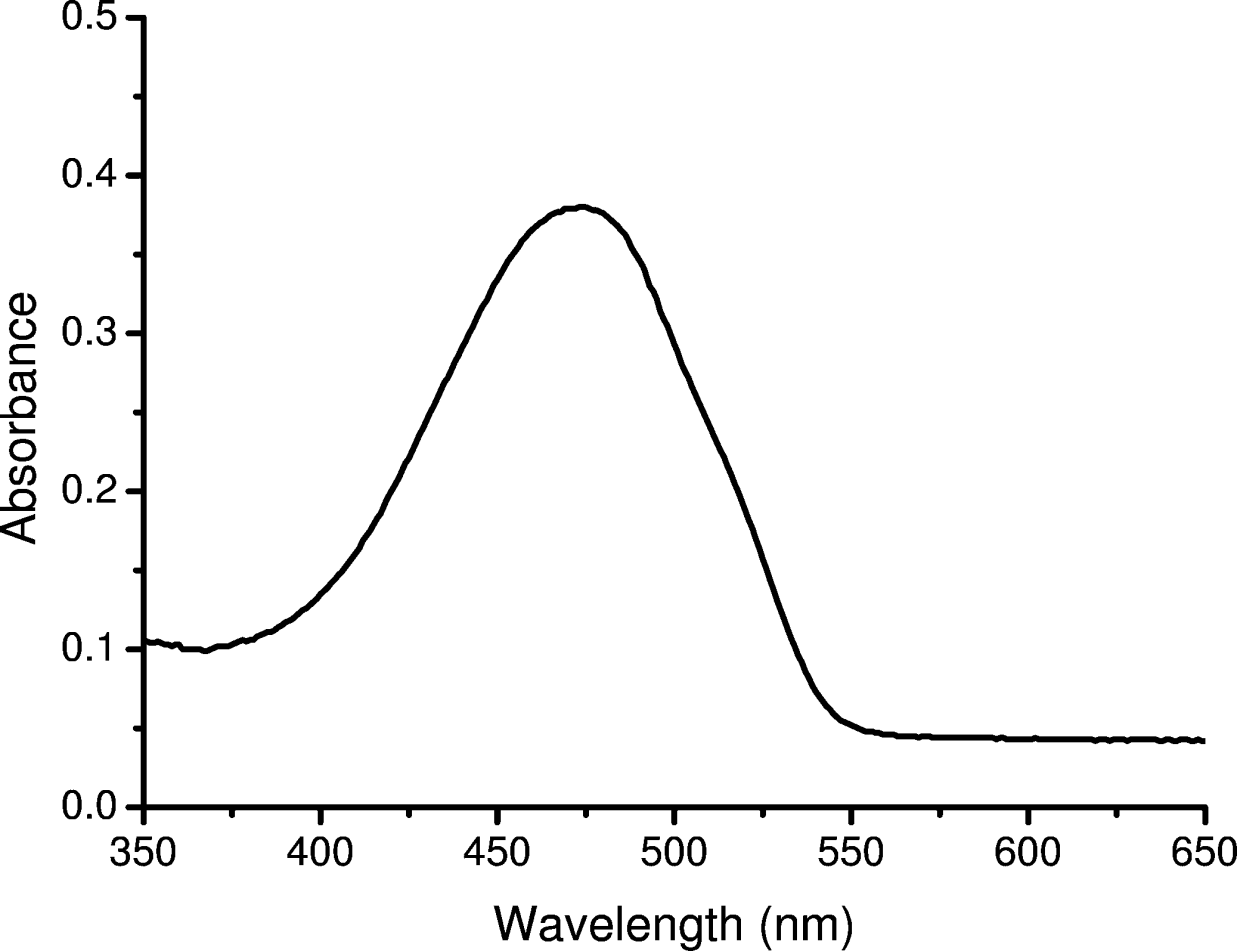
UV–vis spectrum of the title compound in THF solution (1 × 10 ^−5^ *M*).

The UV–vis spectrum and fluorescence spectra of the title compound are shown in Figs. 4 ▸ and 5 ▸, respectively. The UV–vis spectrum is solvent independent. A THF solution of the title compound displays intense broad absorption at 474 nm, which can be assigned to the *n*–π* transition of the imino­pyrrolyl group. The title compound has two emission peaks at 528 nm and 574 nm. It can be seen that the fluorescence intensity of title compound is greatly affected by the solvents. In the polar solvent DMSO, the fluorescence intensity is much weaker than that in the apolar solvent CHCl_3_, which is similar to a previous report (Li *et al.*, 2018 ▸). The title compound shows substantial bathochromic shifts in both absorption and emission when compared to the di­phenyl­boron analogues reported by Gomes and coworkers (Suresh *et al.*, 2012 ▸), which can be ascribed to the planar structure of the title compound.

## Synthesis and crystallization   

To a solution of bis­(pyrrol-2-yl)methyl­enedi­amino­phenyl­ene (1 mmol, 0.32 g) and tri­ethyl­amine (4.2 mmol, 6 mL) in dry di­chloro­methane (15 mL) was slowly added boron trifluoride ethyl ether (7.2 mmol, 2 mL). The resulting solution was stirred overnight, and then saturated potassium carbonate solution was added and stirred for 30 minutes. The resulting solution was extracted and evaporated under vacuum to dryness. The residue was purified by column chromatography eluting with CH_2_Cl_2_ and petroleum ether (*v*:*v* 1:2) to give an orange product, m.p. 435 K. ^1^H NMR (400 MHz, CDCl_3_) *δ* 8.101 (*s*, 2H, =CH–), 7.519 (*s*, 4H, Ar C—H), 6.007 (*s*, 2H, pyrrole CH), 2.412 (*s*, 6H, –CH_3_), 2.270 (*s*, 6H, –CH_3_). HRMS (ESI) *m*/*z*: calculated for C_20_H_20_B_2_F_4_N_4_, (*M* + H)^+^ 415.01521; found 415.01533.

## Refinement   

Crystal data, data collection and structure refinement details are summarized in Table 2 ▸. H atoms were located in a difference-Fourier map, placed in calculated positions (C—H = 0.93 or 0.96 Å) and included in the final cycles of refinement using a riding model, with *U*_iso_(H) = 1.2*U*_eq_(C) or 1.5*U*_eq_(C-meth­yl). Idealized methyl groups were refined as rotating groups.

## Supplementary Material

Crystal structure: contains datablock(s) I. DOI: 10.1107/S2056989021000463/ex2040sup1.cif

Structure factors: contains datablock(s) I. DOI: 10.1107/S2056989021000463/ex2040Isup2.hkl

CCDC reference: 2055687

Additional supporting information:  crystallographic information; 3D view; checkCIF report

## Figures and Tables

**Figure 5:**
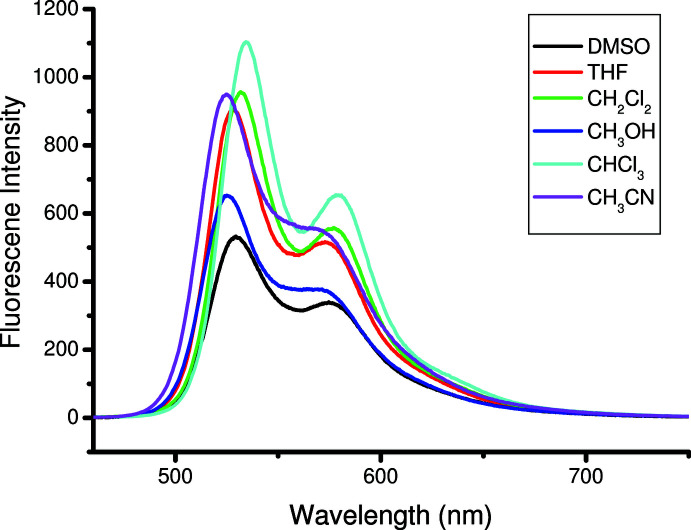
Fluorescence spectra of the title compound in different solutions (1 × 10 ^−5^ *M*).

**Table 1:** Hydrogen-bond geometry (Å, °)

*D*—H⋯*A*	*D*—H	H⋯*A*	*D*⋯*A*	*D*—H⋯*A*
C6—H6*B*⋯F2^i^	0.96	2.49	3.336 (3)	147

Symmetry code: (i) 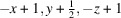.

**Table 2:** Experimental details

Crystal data	
Chemical formula	C_20_H_20_B_2_F_4_N_4_
*M*_r_	414.02
Crystal system, space group	Orthorhombic, *P* *n* *m* *a*
Temperature (K)	110
*a*, *b*, *c* (Å)	20.2495 (9), 6.8046 (5), 13.4969 (5)
*V* (Å^3^)	1859.74 (17)
*Z*	4
Radiation type	Cu *K*α
μ (mm^−1^)	0.99
Crystal size (mm)	0.25 × 0.14 × 0.13

Data collection	
Diffractometer	Rigaku Oxford Diffraction SuperNova, Dual, Cu at zero, AtlasS2
Absorption correction	Multi-scan (*CrysAlis PRO*; Rigaku OD, 2015 ▸)
*T*_min_, *T*_max_	0.478, 1.000
No. of measured, independent and observed [*I* > 2σ(*I*)] reflections	4486, 1815, 1375
*R*_int_	0.046
(sin θ/λ)_max_ (Å^−1^)	0.597

Refinement	
*R*[*F* ^2^ > 2σ(*F* ^2^)], *wR*(*F* ^2^), *S*	0.048, 0.126, 1.01
No. of reflections	1815
No. of parameters	179
No. of restraints	6
H-atom treatment	H-atom parameters constrained
Δρ_max_, Δρ_min_ (e Å^−3^)	0.27, −0.32

Computer programs: *CrysAlis PRO* (Rigaku OD, 2015 ▸), *SHELXT* (Sheldrick, 2015*a* ▸), *SHELXL* (Sheldrick, 2015*b* ▸) and *OLEX2* (Dolomanov *et al.*, 2009 ▸).

## supplementary crystallographic information

### Crystal data

**Table:**

C_20_H_20_B_2_F_4_N_4_	*D*_x_ = 1.479 Mg m^−^^3^
*M**_r_* = 414.02	Cu *K*α radiation, λ = 1.54184 Å
Orthorhombic, *P**n**m**a*	Cell parameters from 1337 reflections
*a* = 20.2495 (9) Å	θ = 3.9–73.0°
*b* = 6.8046 (5) Å	µ = 0.99 mm^−^^1^
*c* = 13.4969 (5) Å	*T* = 110 K
*V* = 1859.74 (17) Å^3^	Block, brown
*Z* = 4	0.25 × 0.14 × 0.13 mm
*F*(000) = 856	

### Data collection

**Table:**

Rigaku Oxford Diffraction SuperNova, Dual, Cu at zero, AtlasS2 diffractometer	1815 independent reflections
Radiation source: micro-focus sealed X-ray tube, SuperNova (Cu) X-ray Source	1375 reflections with *I* > 2σ(*I*)
Mirror monochromator	*R*_int_ = 0.046
Detector resolution: 5.2740 pixels mm^-1^	θ_max_ = 67.1°, θ_min_ = 3.9°
ω scans	*h* = −17→24
Absorption correction: multi-scan (CrysAlisPro; Rigaku OD, 2015)	*k* = −7→8
*T*_min_ = 0.478, *T*_max_ = 1.000	*l* = −13→16
4486 measured reflections	

### Refinement

**Table:**

Refinement on *F*^2^	Primary atom site location: dual
Least-squares matrix: full	Hydrogen site location: inferred from neighbouring sites
*R*[*F*^2^ > 2σ(*F*^2^)] = 0.048	H-atom parameters constrained
*wR*(*F*^2^) = 0.126	*w* = 1/[σ^2^(*F*_o_^2^) + (0.0648*P*)^2^] where *P* = (*F*_o_^2^ + 2*F*_c_^2^)/3
*S* = 1.01	(Δ/σ)_max_ < 0.001
1815 reflections	Δρ_max_ = 0.27 e Å^−^^3^
179 parameters	Δρ_min_ = −0.32 e Å^−^^3^
6 restraints	

### Special details

**Table:**

Geometry. All esds (except the esd in the dihedral angle between two l.s. planes) are estimated using the full covariance matrix. The cell esds are taken into account individually in the estimation of esds in distances, angles and torsion angles; correlations between esds in cell parameters are only used when they are defined by crystal symmetry. An approximate (isotropic) treatment of cell esds is used for estimating esds involving l.s. planes.

### Fractional atomic coordinates and isotropic or equivalent isotropic displacement parameters (Å^2^)

**Table:**

	*x*	*y*	*z*	*U*_iso_*/*U*_eq_	Occ. (<1)
F1	0.47574 (6)	0.5830 (2)	0.79642 (10)	0.0251 (3)	
F2	0.27560 (6)	0.58327 (19)	0.39551 (9)	0.0214 (3)	
N1	0.57949 (12)	0.750000	0.83332 (18)	0.0181 (6)	
N2	0.54122 (11)	0.750000	0.66808 (19)	0.0152 (5)	
N3	0.37529 (11)	0.750000	0.33323 (18)	0.0146 (5)	
N4	0.27854 (11)	0.750000	0.2358 (2)	0.0167 (6)	
C1	0.63149 (14)	0.750000	0.7670 (2)	0.0175 (6)	
C2	0.60426 (15)	0.750000	0.9262 (2)	0.0196 (7)	
C3	0.67356 (15)	0.750000	0.9190 (2)	0.0215 (7)	
H3	0.702742	0.750000	0.972227	0.026*	
C4	0.69141 (14)	0.750000	0.8194 (2)	0.0183 (6)	
C5	0.56119 (16)	0.750000	1.0158 (2)	0.0256 (7)	
H5A	0.550754	0.617060	1.033709	0.038*	0.5
H5B	0.521163	0.820249	1.001678	0.038*	0.5
H5C	0.583911	0.812692	1.069602	0.038*	0.5
C6	0.76010 (14)	0.750000	0.7777 (3)	0.0223 (7)	
H6A	0.788406	0.827009	0.819538	0.034*	0.5
H6B	0.759521	0.805418	0.712325	0.034*	0.5
H6C	0.776318	0.617573	0.774672	0.034*	0.5
C7	0.60731 (14)	0.750000	0.6711 (2)	0.0174 (6)	
H7	0.633991	0.750000	0.615007	0.021*	
C8	0.50243 (14)	0.750000	0.5815 (2)	0.0153 (6)	
C9	0.43380 (14)	0.750000	0.5922 (2)	0.0190 (7)	
H9	0.415253	0.750000	0.655206	0.023*	
C10	0.39326 (14)	0.750000	0.5099 (2)	0.0212 (7)	
H10	0.347688	0.750000	0.518466	0.025*	
C11	0.41924 (14)	0.750000	0.4141 (2)	0.0150 (6)	
C12	0.48845 (14)	0.750000	0.4035 (2)	0.0169 (6)	
H12	0.507081	0.750000	0.340566	0.020*	
C13	0.52863 (13)	0.750000	0.4856 (2)	0.0183 (7)	
H13	0.574212	0.750000	0.477137	0.022*	
C14	0.39064 (13)	0.750000	0.2381 (2)	0.0172 (6)	
H14	0.433769	0.750000	0.214397	0.021*	
C15	0.33467 (13)	0.750000	0.1766 (2)	0.0169 (6)	
C16	0.31589 (16)	0.750000	0.0770 (2)	0.0199 (7)	
C17	0.24674 (15)	0.750000	0.0784 (2)	0.0208 (7)	
H17	0.219502	0.750000	0.022896	0.025*	
C18	0.22542 (14)	0.750000	0.1769 (2)	0.0192 (7)	
C19	0.35899 (15)	0.750000	−0.0125 (2)	0.0260 (7)	
H19A	0.358113	0.622295	−0.042734	0.039*	0.5
H19B	0.403441	0.781639	0.006382	0.039*	0.5
H19C	0.343184	0.846066	−0.058910	0.039*	0.5
C20	0.15647 (15)	0.750000	0.2183 (3)	0.0276 (8)	
H20A	0.138964	0.881066	0.216494	0.041*	0.5
H20B	0.157389	0.704099	0.285535	0.041*	0.5
H20C	0.129033	0.664835	0.179283	0.041*	0.5
B1	0.51243 (16)	0.750000	0.7788 (2)	0.0164 (7)	
B2	0.29629 (15)	0.750000	0.3467 (3)	0.0163 (7)	

### Atomic displacement parameters (Å^2^)

**Table:**

	*U*^11^	*U*^22^	*U*^33^	*U*^12^	*U*^13^	*U*^23^
F1	0.0203 (6)	0.0351 (8)	0.0197 (6)	−0.0089 (6)	−0.0011 (5)	0.0052 (6)
F2	0.0177 (6)	0.0275 (7)	0.0190 (6)	−0.0052 (5)	0.0007 (5)	0.0037 (6)
N1	0.0159 (12)	0.0255 (14)	0.0128 (12)	0.000	−0.0019 (10)	0.000
N2	0.0108 (10)	0.0211 (13)	0.0137 (12)	0.000	−0.0022 (10)	0.000
N3	0.0109 (11)	0.0196 (12)	0.0133 (12)	0.000	0.0000 (10)	0.000
N4	0.0114 (11)	0.0199 (13)	0.0187 (13)	0.000	−0.0012 (10)	0.000
C1	0.0144 (13)	0.0195 (15)	0.0186 (15)	0.000	−0.0019 (12)	0.000
C2	0.0233 (15)	0.0194 (15)	0.0163 (15)	0.000	−0.0034 (13)	0.000
C3	0.0203 (15)	0.0230 (16)	0.0213 (15)	0.000	−0.0064 (13)	0.000
C4	0.0147 (13)	0.0166 (14)	0.0235 (15)	0.000	−0.0078 (12)	0.000
C5	0.0239 (14)	0.0358 (19)	0.0169 (15)	0.000	−0.0045 (13)	0.000
C6	0.0140 (13)	0.0262 (17)	0.0267 (17)	0.000	−0.0062 (13)	0.000
C7	0.0115 (13)	0.0221 (15)	0.0185 (15)	0.000	−0.0007 (12)	0.000
C8	0.0136 (13)	0.0182 (14)	0.0142 (15)	0.000	−0.0024 (11)	0.000
C9	0.0136 (13)	0.0309 (17)	0.0126 (14)	0.000	0.0015 (12)	0.000
C10	0.0099 (12)	0.0345 (17)	0.0193 (16)	0.000	0.0000 (12)	0.000
C11	0.0128 (13)	0.0179 (14)	0.0144 (14)	0.000	−0.0024 (11)	0.000
C12	0.0123 (13)	0.0259 (15)	0.0124 (14)	0.000	0.0029 (11)	0.000
C13	0.0085 (12)	0.0255 (16)	0.0209 (16)	0.000	−0.0008 (12)	0.000
C14	0.0118 (13)	0.0200 (15)	0.0198 (15)	0.000	−0.0010 (12)	0.000
C15	0.0119 (12)	0.0179 (14)	0.0209 (16)	0.000	0.0006 (12)	0.000
C16	0.0213 (14)	0.0212 (15)	0.0173 (14)	0.000	−0.0064 (13)	0.000
C17	0.0196 (14)	0.0264 (17)	0.0166 (15)	0.000	−0.0093 (13)	0.000
C18	0.0158 (14)	0.0235 (16)	0.0183 (15)	0.000	−0.0044 (12)	0.000
C19	0.0229 (15)	0.0370 (18)	0.0182 (16)	0.000	−0.0004 (13)	0.000
C20	0.0148 (14)	0.045 (2)	0.0234 (17)	0.000	−0.0037 (13)	0.000
B1	0.0160 (15)	0.0232 (18)	0.0100 (16)	0.000	−0.0018 (13)	0.000
B2	0.0092 (14)	0.0238 (18)	0.0158 (15)	0.000	−0.0036 (13)	0.000

### Geometric parameters (Å, º)

**Table:**

F1—B1	1.378 (2)	C6—H6C	0.9600
F2—B2	1.377 (2)	C7—H7	0.9300
N1—C1	1.382 (4)	C8—C9	1.397 (4)
N1—C2	1.350 (4)	C8—C13	1.399 (4)
N1—B1	1.544 (4)	C9—H9	0.9300
N2—C7	1.339 (4)	C9—C10	1.381 (4)
N2—C8	1.408 (4)	C10—H10	0.9300
N2—B1	1.604 (4)	C10—C11	1.396 (4)
N3—C11	1.408 (4)	C11—C12	1.409 (4)
N3—C14	1.321 (4)	C12—H12	0.9300
N3—B2	1.610 (4)	C12—C13	1.374 (4)
N4—C15	1.389 (4)	C13—H13	0.9300
N4—C18	1.338 (4)	C14—H14	0.9300
N4—B2	1.538 (4)	C14—C15	1.404 (4)
C1—C4	1.404 (4)	C15—C16	1.398 (4)
C1—C7	1.384 (4)	C16—C17	1.400 (4)
C2—C3	1.407 (4)	C16—C19	1.491 (5)
C2—C5	1.491 (5)	C17—H17	0.9300
C3—H3	0.9300	C17—C18	1.398 (5)
C3—C4	1.393 (5)	C18—C20	1.504 (4)
C4—C6	1.500 (4)	C19—H19A	0.9600
C5—H5A	0.9600	C19—H19B	0.9600
C5—H5B	0.9600	C19—H19C	0.9600
C5—H5C	0.9600	C20—H20A	0.9600
C6—H6A	0.9600	C20—H20B	0.9600
C6—H6B	0.9600	C20—H20C	0.9600
			
C1—N1—B1	111.2 (2)	N3—C11—C12	123.4 (3)
C2—N1—C1	108.6 (2)	C10—C11—N3	118.7 (2)
C2—N1—B1	140.2 (3)	C10—C11—C12	117.9 (3)
C7—N2—C8	125.6 (3)	C11—C12—H12	119.7
C7—N2—B1	109.6 (2)	C13—C12—C11	120.5 (3)
C8—N2—B1	124.8 (2)	C13—C12—H12	119.7
C11—N3—B2	122.7 (2)	C8—C13—H13	119.3
C14—N3—C11	127.2 (2)	C12—C13—C8	121.4 (3)
C14—N3—B2	110.1 (2)	C12—C13—H13	119.3
C15—N4—B2	111.6 (2)	N3—C14—H14	123.7
C18—N4—C15	108.4 (3)	N3—C14—C15	112.6 (3)
C18—N4—B2	140.0 (3)	C15—C14—H14	123.7
N1—C1—C4	109.4 (3)	N4—C15—C14	108.7 (3)
N1—C1—C7	109.6 (2)	N4—C15—C16	109.3 (2)
C7—C1—C4	140.9 (3)	C16—C15—C14	142.0 (3)
N1—C2—C3	107.9 (3)	C15—C16—C17	105.0 (3)
N1—C2—C5	122.4 (3)	C15—C16—C19	128.4 (3)
C3—C2—C5	129.8 (3)	C17—C16—C19	126.6 (3)
C2—C3—H3	125.5	C16—C17—H17	125.6
C4—C3—C2	109.0 (3)	C18—C17—C16	108.7 (3)
C4—C3—H3	125.5	C18—C17—H17	125.6
C1—C4—C6	127.8 (3)	N4—C18—C17	108.5 (3)
C3—C4—C1	105.2 (3)	N4—C18—C20	121.7 (3)
C3—C4—C6	127.1 (3)	C17—C18—C20	129.8 (3)
C2—C5—H5A	109.5	C16—C19—H19A	109.5
C2—C5—H5B	109.5	C16—C19—H19B	109.5
C2—C5—H5C	109.5	C16—C19—H19C	109.5
H5A—C5—H5B	109.5	H19A—C19—H19B	109.5
H5A—C5—H5C	109.5	H19A—C19—H19C	109.5
H5B—C5—H5C	109.5	H19B—C19—H19C	109.5
C4—C6—H6A	109.5	C18—C20—H20A	109.5
C4—C6—H6B	109.5	C18—C20—H20B	109.5
C4—C6—H6C	109.5	C18—C20—H20C	109.5
H6A—C6—H6B	109.5	H20A—C20—H20B	109.5
H6A—C6—H6C	109.5	H20A—C20—H20C	109.5
H6B—C6—H6C	109.5	H20B—C20—H20C	109.5
N2—C7—C1	112.5 (3)	F1—B1—F1^i^	111.1 (3)
N2—C7—H7	123.8	F1—B1—N1	113.08 (16)
C1—C7—H7	123.8	F1^i^—B1—N1	113.08 (16)
C9—C8—N2	118.0 (3)	F1—B1—N2	110.87 (17)
C9—C8—C13	118.2 (3)	F1^i^—B1—N2	110.87 (17)
C13—C8—N2	123.8 (3)	N1—B1—N2	97.1 (2)
C8—C9—H9	119.7	F2—B2—F2^i^	110.9 (2)
C10—C9—C8	120.6 (3)	F2—B2—N3	110.86 (15)
C10—C9—H9	119.7	F2^i^—B2—N3	110.86 (15)
C9—C10—H10	119.3	F2—B2—N4	113.23 (15)
C9—C10—C11	121.4 (3)	F2^i^—B2—N4	113.23 (15)
C11—C10—H10	119.3	N4—B2—N3	97.0 (2)
			
N1—C1—C4—C3	0.000 (1)	C11—N3—C14—C15	180.000 (1)
N1—C1—C4—C6	180.000 (1)	C11—N3—B2—F2^i^	61.82 (18)
N1—C1—C7—N2	0.000 (1)	C11—N3—B2—F2	−61.81 (18)
N1—C2—C3—C4	0.000 (1)	C11—N3—B2—N4	180.000 (1)
N2—C8—C9—C10	180.000 (1)	C11—C12—C13—C8	0.000 (1)
N2—C8—C13—C12	180.000 (1)	C13—C8—C9—C10	0.000 (1)
N3—C11—C12—C13	180.000 (1)	C14—N3—C11—C10	180.000 (1)
N3—C14—C15—N4	0.000 (1)	C14—N3—C11—C12	0.000 (1)
N3—C14—C15—C16	180.000 (1)	C14—N3—B2—F2	118.19 (18)
N4—C15—C16—C17	0.000 (1)	C14—N3—B2—F2^i^	−118.18 (18)
N4—C15—C16—C19	180.000 (1)	C14—N3—B2—N4	0.000 (1)
C1—N1—C2—C3	0.000 (1)	C14—C15—C16—C17	180.000 (1)
C1—N1—C2—C5	180.000 (1)	C14—C15—C16—C19	0.000 (1)
C1—N1—B1—F1^i^	−116.3 (2)	C15—N4—C18—C17	0.000 (1)
C1—N1—B1—F1	116.3 (2)	C15—N4—C18—C20	180.000 (1)
C1—N1—B1—N2	0.000 (1)	C15—N4—B2—F2^i^	116.33 (18)
C2—N1—C1—C4	0.000 (1)	C15—N4—B2—F2	−116.32 (18)
C2—N1—C1—C7	180.000 (1)	C15—N4—B2—N3	0.000 (1)
C2—N1—B1—F1	−63.7 (2)	C15—C16—C17—C18	0.000 (1)
C2—N1—B1—F1^i^	63.7 (2)	C16—C17—C18—N4	0.000 (1)
C2—N1—B1—N2	180.000 (1)	C16—C17—C18—C20	180.000 (1)
C2—C3—C4—C1	0.000 (1)	C18—N4—C15—C14	180.000 (1)
C2—C3—C4—C6	180.000 (1)	C18—N4—C15—C16	0.000 (1)
C4—C1—C7—N2	180.000 (1)	C18—N4—B2—F2^i^	−63.67 (18)
C5—C2—C3—C4	180.000 (1)	C18—N4—B2—F2	63.68 (18)
C7—N2—C8—C9	180.000 (1)	C18—N4—B2—N3	180.000 (1)
C7—N2—C8—C13	0.000 (1)	C19—C16—C17—C18	180.0
C7—N2—B1—F1^i^	118.07 (18)	B1—N1—C1—C4	180.000 (1)
C7—N2—B1—F1	−118.07 (18)	B1—N1—C1—C7	0.000 (1)
C7—N2—B1—N1	0.000 (1)	B1—N1—C2—C3	180.000 (1)
C7—C1—C4—C3	180.000 (1)	B1—N1—C2—C5	0.000 (1)
C7—C1—C4—C6	0.000 (1)	B1—N2—C7—C1	0.000 (1)
C8—N2—C7—C1	180.000 (1)	B1—N2—C8—C9	0.000 (1)
C8—N2—B1—F1^i^	−61.93 (18)	B1—N2—C8—C13	180.000 (1)
C8—N2—B1—F1	61.93 (18)	B2—N3—C11—C10	0.000 (1)
C8—N2—B1—N1	180.000 (1)	B2—N3—C11—C12	180.000 (1)
C8—C9—C10—C11	0.000 (1)	B2—N3—C14—C15	0.000 (1)
C9—C8—C13—C12	0.000 (1)	B2—N4—C15—C14	0.000 (1)
C9—C10—C11—N3	180.000 (1)	B2—N4—C15—C16	180.000 (1)
C9—C10—C11—C12	0.000 (1)	B2—N4—C18—C17	180.000 (1)
C10—C11—C12—C13	0.000 (1)	B2—N4—C18—C20	0.000 (1)

Symmetry code: (i) *x*, −*y*+3/2, *z*.

### Hydrogen-bond geometry (Å, º)

**Table:**

*D*—H···*A*	*D*—H	H···*A*	*D*···*A*	*D*—H···*A*
C6—H6*B*···F2^ii^	0.96	2.49	3.336 (3)	147

Symmetry code: (ii) −*x*+1, *y*+1/2, −*z*+1.
